# A bibliometric analysis of artificial intelligence applied to cervical cancer

**DOI:** 10.3389/fmed.2025.1562818

**Published:** 2025-04-08

**Authors:** Qiang Huang, Wenmei Su, Shujun Li, Yanming Lin, Zhen Cheng, Yuting Chen, Yanli Mo

**Affiliations:** ^1^Department of Ultrasound, Affiliated Hospital of Guangdong Medical University, Zhanjiang, China; ^2^Department of Pulmonary Oncology, Affiliated Hospital of Guangdong Medical University, Zhanjiang, China

**Keywords:** cervical cancer, artificial intelligence, AI, bibliometric analysis, machine learning

## Abstract

**Objective:**

This study conducts a bibliometric analysis of artificial intelligence (AI) applications in cervical cancer to provide a comprehensive overview of the research landscape and current advancements.

**Methods:**

Relevant publications on cervical cancer and AI were retrieved from the Web of Science Core Collection. Bibliometric analysis was performed using CiteSpace and VOSviewer to assess publication trends, authorship, country and institutional contributions, journal sources, and keyword co-occurrence patterns.

**Results:**

From 1996 to 2024, our analysis of 770 publications on cervical cancer and AI showed a surge in research, with 86% published in the last 5 years. China (315 pubs, 32%) and the US (155 pubs, 16%) were the top contributors. Key institutions were the Chinese Academy of Sciences, Southern Medical University, and Huazhong University of Science and Technology. Research hotspots included disease prediction, image analysis, and machine learning in cervical cancer. Schiffman led in publications (12) and citations (207). China had the highest citations (3,819). Top journals were “Diagnostics,” “Scientific Reports,” and “Frontiers in Oncology.” Keywords like “machine learning” and “deep learning” indicated current research trends. This study maps the field's growth, highlighting key contributors and topics.

**Conclusion:**

This bibliometric analysis provides valuable insights into research trends and hotspots, guiding future studies and fostering collaboration to enhance AI applications in cervical cancer.

## Introduction

Cervical cancer is the fourth most common malignancy among women globally, posing a significant threat to women's health. Despite advances in human papillomavirus (HPV) screening and vaccination programs that have reduced disease incidence, the high prevalence and severity of cervical cancer persist. The prognosis for patients with intermediate and advanced stages of cervical cancer remains varied (1), and treatment challenges continue. Moreover, currently there is a shortage of pathologists and uneven resource distribution, and existing screening methods such as cervical cytology screening and HPV testing have limitations (2, 3). Over recent decades, the management of cervical cancer has made significant progress, driven by advancements in early diagnosis, surgical techniques, and adjuvant therapies. Future research will prioritize personalized treatment paradigms and exploration of novel therapeutic strategies to further improve prognostic outcomes (4).

Artificial intelligence (AI) holds great potential for improving diagnosis, treatment, and prognosis across various fields, especially in cancer research. As medical data volumes grow and computing power advances, integrating AI into cervical cancer research offers promising opportunities to enhance diagnostic efficiency and broaden screening reach. The DeepGEM method's AUC scores, ranging from 0.82 to 0.96, demonstrate AI's transformative predictive accuracy in personalized oncology (5).

Recent developments in deep learning algorithms have shown remarkable success in image recognition. Given that cervical cancer screening relies heavily on the analysis of cervical cell and histopathological images, AI can learn from large datasets to identify disease characteristics, improving diagnostic accuracy (6, 7). For instance, researchers developed an AI-assisted TBS (AIATBS) system for analyzing digital cervical liquid-based cytology slides, demonstrating superior sensitivity compared to experienced cytologists (8). Other studies employing deep learning for Pap smear and colposcopy image analysis have also improved screening accuracy by correctly classifying images and identifying high-grade lesions (9, 10). Furthermore, Miyagi et al.'s study (11) demonstrates the potential of AI in the clinic for sorting cervical squamous intraepithelial lesions (SIL) and human papillomavirus (HPV) types in colposcopy images. Prior studies underscore the growing role of AI in enhancing the precision of cervical cancer diagnosis, which is crucial for personalized treatment planning and patient management. Xiaohui Zhu et al. developed a cervical cancer AI model utilizing artificial neural networks (ANN), showing high accuracy in predicting staging, histology, grading, and lymph node metastasis (LNM) (12). This model aids clinicians in making timely and precise diagnostic decisions, ultimately enhancing patient outcomes.

Despite the promising potential of AI in cervical cancer, challenges remain. Although research on AI applications in cervical cancer is increasing, there is a lack of comprehensive insights into the overall field. Studies often appear fragmented, lacking a unified understanding of key research directions, hotspots, and trends. Additionally, quality and quantity issues in data continue to pose significant obstacles.

This study endeavors to provide novice researchers with a panoramic perspective on the role of AI in cervical cancer, pinpointing pivotal research hotspots, and delving into prospective research avenues via an exhaustive bibliometric analysis.

## Materials and methods

### Data sources and search strategy

The Web of Science (WoS) Core Collection (https://www.webofscience.com) is a renowned international database for academic literature, providing comprehensive abstracts and indexing across numerous disciplines. It includes a wide range of journals (including conference proceedings), unique citation index capabilities, and robust analytical tools. The WoS enables users to refine high-impact papers through various dimensions, such as research direction, source publication, author, institution, and publication year.

The study focused on the Science Citation Index Expanded (SCIE) and Emerging Sources Citation Index (ESCI) within the WoS Core Collection, which encompass over 10,000 prestigious journals and millions of publications across multiple fields of natural sciences. We conducted searches in the SCIE and ESCI databases from January 1, 1990, to August 16, 2024, using the following search query: (TS=(“artificial intelligence”) OR TS=(“transfer learning”) OR TS=(“AI”) OR TS=(“neural network”) OR TS=(“machine learning”) OR TS=(“deep learning”)) AND (TS=(“cervical neoplasm”) OR TS=(“cervical cancer”) OR TS=(“cervical tumor”) OR TS=(“cervical carcinoma”)).

This search returned a total of 1,025 research papers. After filtering for language, we narrowed this to 906 English-language articles. We manually screened titles and abstracts to determine relevance, resulting in the removal of 136 unrelated publications. Ultimately, this study included 770 papers for bibliometric and visual analysis. Our analysis focused on research trends and hotspots, assessing indicators such as authorship, countries, institutions, citations, journals, and keywords.

### Bibliometric analysis

The full records and cited references for the 770 articles retrieved from WoSCC were exported in plain text format. We employed VOSviewer (RRID:SCR_023516) software for bibliometric analysis and visualization. VOSviewer offers robust capabilities for visualizing networks of authors, institutions, and literature co-citation, helping researchers quickly understand the structure and connections within the field. Additionally, it is vital for identifying research hotspots and trends (13).

CiteSpace (RRID:SCR_025121) is another citation visualization tool, specialized in analyzing knowledge within scientific literature. This software effectively illustrates the main research directions and core topics in the field, along with their interrelationships, thus aiding researchers in comprehensively assessing the status of a specific area, including hotspots, emerging trends, leading authors, and institutions (14).

We imported the data into VOSviewer (version 1.6.20) and CiteSpace (version 6.3.1) for literature data analysis and visualization. Specific analyses conducted included cooperation network analysis, co-occurrence analysis, bibliographic coupling, and co-citation analysis. The units of analysis included authors, countries, institutions, journals, and keywords. We then adjusted the screening thresholds based on the output, resulting in the following criteria:

Author: 5 papers
Cited Author: more than 35 citations
Country: 5 papers
Institution: 6 papers
Citation: over 25 citations
Journal: 5 papers
Keyword: at least 20 occurrences

## Results and analysis

### Distribution of literature publication years

**Figure 1:**
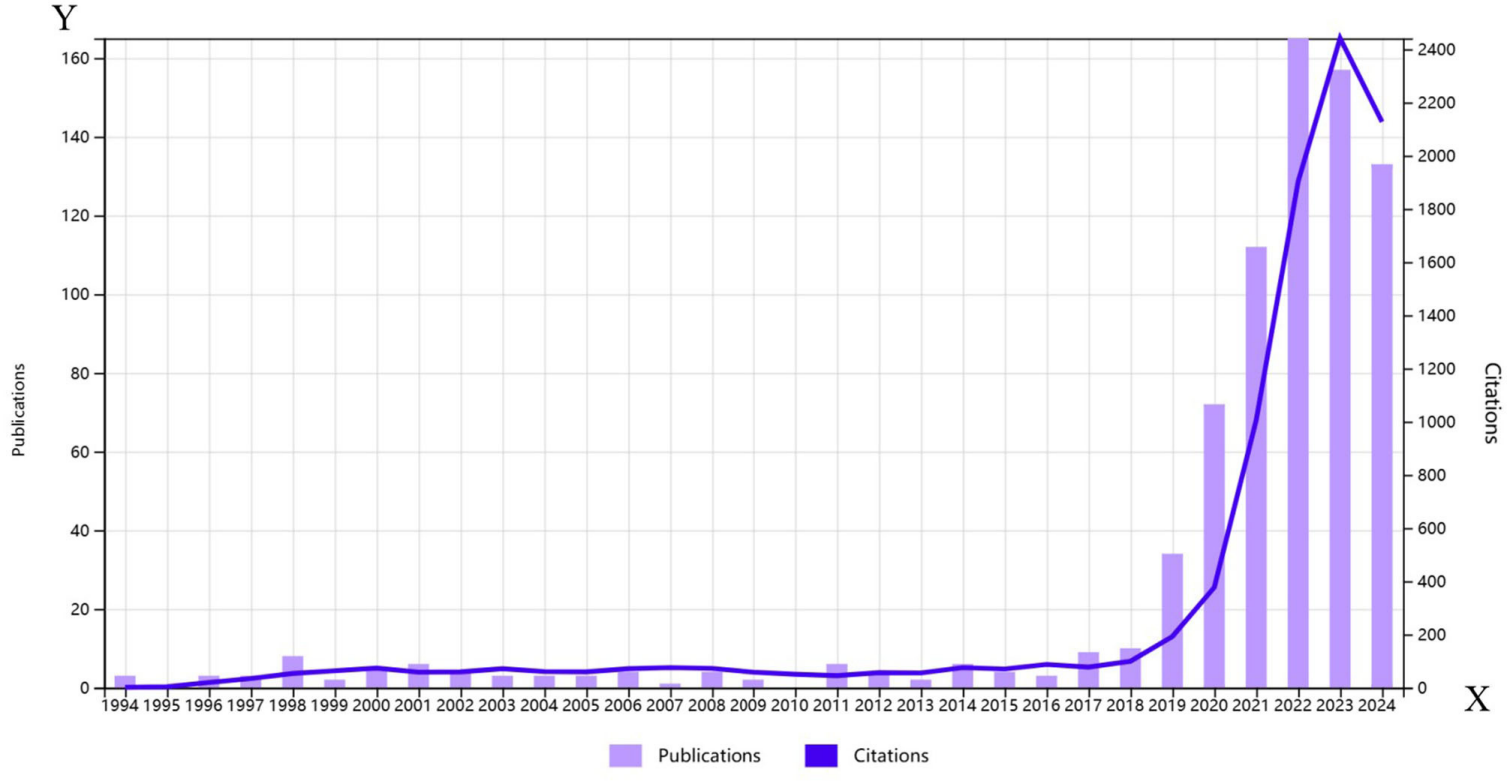
Annual global publication and citation trends of artificial intelligence in cervical cancer from 1990 to 2024.

The bibliometric analysis covered 770 publications from January 1, 1996, to August 16, 2024, sourced from the SCI Expanded and ESCI indexes. According to the WoS citation report, global publications and citation trends are illustrated in Figure 1. Prior to 2019, publication numbers were relatively low, but the last 5 years saw a significant surge, with recent years accounting for 86% of total publications (35 in 2019, 74 in 2020, 117 in 2021, 160 in 2022, 156 in 2023, and 120 in 2024, totaling 662). The decline in 2024 may be due to the cut-off time being less than a year. Overall, the rapid increase in research papers on cervical cancer and AI indicates growing interest and investment in this area, likely driven by advancements in computing resources and algorithms.

### Author analysis

This study conducted an author analysis of the literature, focusing on the number of papers published, citations, and H-index, using visualization tools to create an author cooperation network. A total of 4,441 authors and 15,959 cited authors contributed to this research area. Author Schiffman leads in both publication count (12 papers) and citations (207). Antani Sameer follows with 10 papers and 144 citations, while Befano, Brian, Chen, and Lv tie with 9 papers each. Their productivity underscores their significant influence in this field. Table 1 lists the top 10 authors based on publication and citation metrics.

**Table 1:** The top 10 productive authors and the top 10 co-cited authors.

**Rank**	**Author**	**Documents**	**H-index**	**Citation**	**Country**	**TLS**	**Cite-author**	**Citations**	**TLS**
1	Schiffman, Mark	12	24	207	USA	55	Jemal, A	126	462
2	Antani, Sameer	10	36	144	USA	46	He, Km	113	719
3	Befano, Brian	9	19	101	USA	50	Zhang, L	111	788
4	Chen, Chen	9	9	85	China	19	Arbyn, M	107	435
5	Lv, Xiaoyi	9	25	85	USA	19	Sung, H	88	322
6	Egemen, Didem	8	19	75	USA	41	Szegedy, C	82	554
7	Wang, Wei	8	9	52	China	0	Plissiti, Me	79	538
8	Chen, Cheng	7	5	36	China	15	Ronneberger, O	79	323
9	De Sanjose, Silvia	7	87	63	USA	40	Schiffman, M	57	238
10	Albuquerque, Kevin	6	20	197	USA	0	Xue, P	56	210

TLS: Total Link Strength.

The H-index is a vital indicator in bibliometric analysis, reflecting both productivity and impact. Antani Sameer's H-index of 36 signifies his substantial influence in this research domain. Additionally, betweenness centrality (BC) value reveals each author's role in the network. A BC value exceeding 0.1 indicates key nodes. Citespace analysis reveals that Unknown (BC = 0.26) ranks first, followed by Jamal A (0.16), Ronneberger O (0.13), and He Km (0.11), highlighting these scholars as pivotal in academic exchanges and fostering collaboration.

### Country analysis

**Figure 2:**
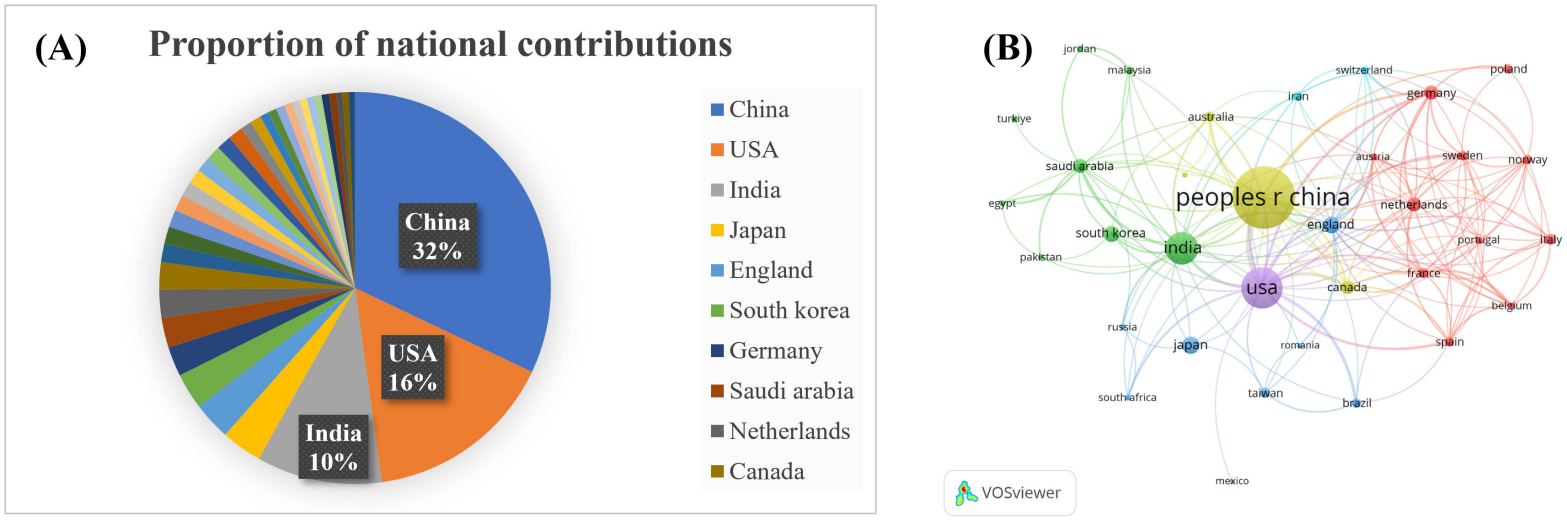
Analysis of countries. **(A)** Proportion of national contributions; **(B)** Clustering of collaboration among countries. Larger nodes indicate more publications.

Country distribution is a critical aspect of this bibliometric analysis. We analyzed the publishing activity of 80 countries in the literature on cervical cancer and AI. Table 2 lists the top 10 countries, while Figure 2A illustrates the share of publications per country, and Figure 2B shows the national cooperation network. China leads with 315 publications (32%), followed by the United States with 155 articles (16%) and India with 102 (10%). Together, these countries represent 50% of total publications. China has the highest citation count (3,819), followed by the United States (2,825) and India (1,062), showcasing robust research capabilities, particularly from developing countries.

**Table 2:** The top 10 co-cited references.

**Rank**	**Title**	**Total Citations**	**Average per Year**	**Author**	**Source Title**	**Publication Year**
1	Cervical cancer classification using convolutional neural networks and extreme learning machines	130	26	Ghoneim, Ahmed	Computers in biology and medicine	2020
2	Deep convolutional neural network with transfer learning for rectum toxicity prediction in cervical cancer radiotherapy: a feasibility study	117	14.63	Zhen, Xin	Physics in medicine and biology	2017
3	Inception v3 based cervical cell classification combined with artificially extracted features	109	21.8	Dong, N	Applied soft computing	2020
4	DeepCervix: a deep learning-based framework for the classification of cervical cells using hybrid deep feature fusion techniques	96	24	Rahaman	Computers in biology and medicine	2021
5	Computer-assisted cervical-cancer screening using neural networks	95	3.06	Mango, LJ	Cancer letters	1994
6	Survival outcome prediction in cervical cancer: cox models vs deep-learning model	93	15.5	Matsuo, Koji	American journal of obstetrics and gynecology	2019
7	Machine learning for assisting cervical cancer diagnosis: an ensemble approach	80	16	Lu, Jiayi	Future generation computer systems-the international journal of escience	2020
8	Diagnosis of Cervical Cancer based on Ensemble Deep Learning Network using Colposcopy Images	75	18.75	Chandran	Biomed research international	2021
9	Detection of cervical cancer cells based on strong feature CNN-SVM network	74	14.8	Jia, A	Neurocomputing	2020
10	Intelligent inverse treatment planning via deep reinforcement learning, a proof-of-principle study in high dose-rate brachytherapy for cervical cancer	74	12.33	Shen, Chenyang	Physics in medicine and biology	2019

The BC value is essential for understanding the role of countries within the research network. Analysis indicates that Unknown (BC = 0.26) ranks highest, with Jamal A (0.16), Ronneberger O (0.13), and He Km (0.11) as significant contributors to the exchange of knowledge.

Total link strength (TLS) is a measure of a country's status in the global research network. The leading countries by TLS are the United States (116), China (80), and India (51). The United States also holds the highest BC value (0.85), indicating strong research investments and efficient resource integration. This analysis highlights an uneven global development trend in cervical cancer AI research, with the United States and China at the forefront. Enhanced international collaboration will be crucial for advancing research in this field.

### Institution analysis

The evolution of AI in cervical cancer research has involved contributions from 1,419 institutions among the 770 analyzed publications. Table 2 lists the top ten institutions, with eight from China and the top three being Chinese. This reflects China's strong commitment to AI in cervical cancer research. The Chinese Academy of Sciences leads with 21 publications, supported by top experts and advanced facilities, highlighting its significant academic influence. Southern Medical University follows with 19 papers, and Huazhong University of Science and Technology has produced 17. The citation counts for these institutions indicate a positive correlation between publication volume and citation influence.

**Figure 3:**
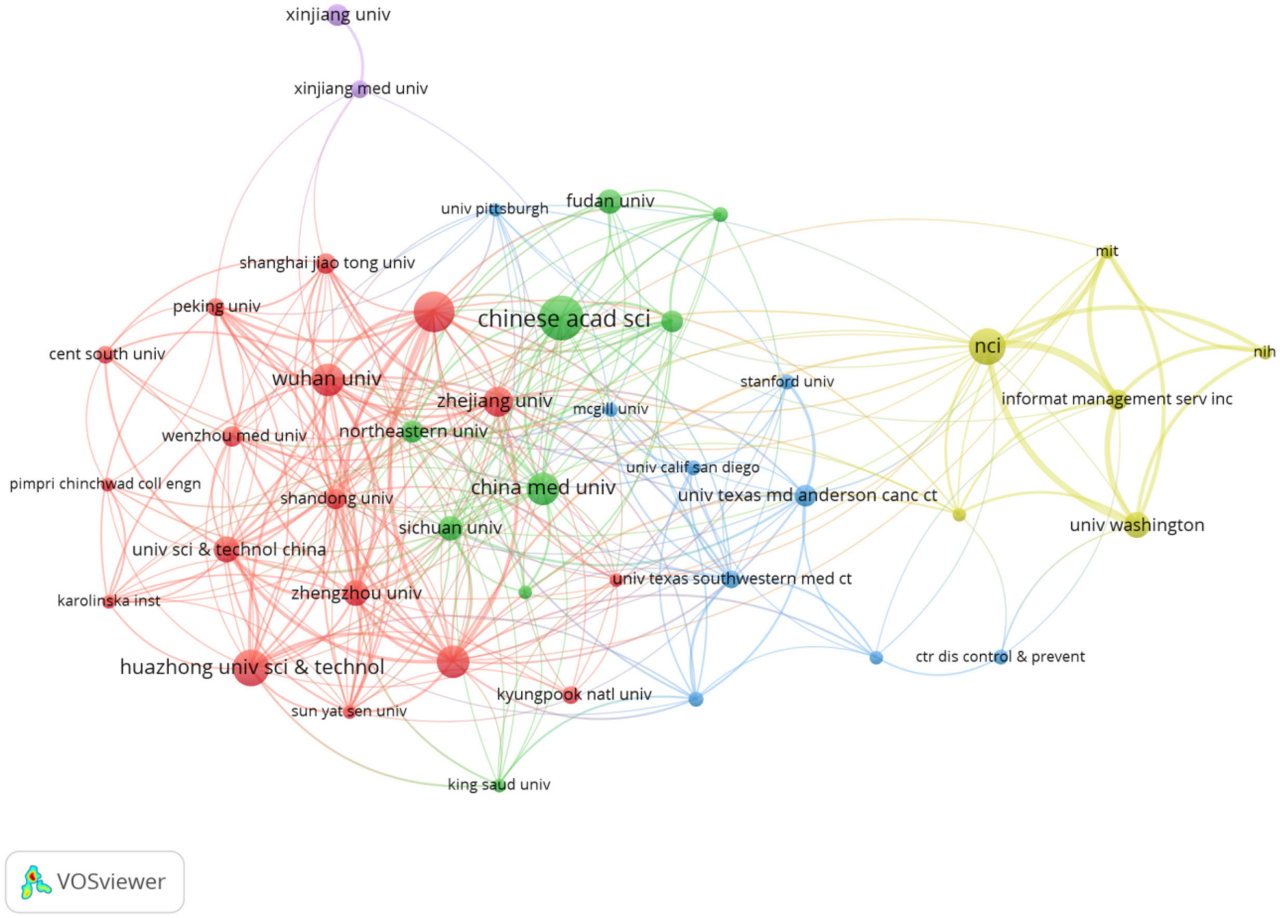
Cooperation between institutions.

Analysis of the institutional cooperation network in Figure 3 illustrates limited international collaboration, mostly confined within the same country. This emphasizes the urgent need for enhanced interdisciplinary and international cooperation. Moving forward, institutions should bolster exchanges, prioritize interdisciplinary integration, and focus on international partnerships to elevate research quality and impact in cervical cancer AI.

### Citation analysis

Citation analysis provides insights into academic influence and knowledge flow. An analysis of 770 works from WOSCC identified 21,694 references, with 31 publications cited at least 25 times. The significance of relevant literature is assessed through metrics like the H-index. Table 3 lists the top 10 most cited works, with Ghoneim et al.'s (15) publication on classification using convolutional neural networks receiving the highest citation (130) and an H-index of 21 (15). This study employed transfer learning alongside a convolutional neural network model and an extreme learning machine (ELM) classifier to analyze cervical cancer images.

**Table 3:** The top 10 journals and the top 10 cited-journals.

**Journal**	**Documents**	**JCR**	**IF(5)**	**Cited-journal**	**Citations**	**JCR**
Diagnostics	25	Q1	3	Med Phys	644	Q1
Scientific Reports	25	Q1	3.8	Int J Radiat Oncol	575	Q2
Frontiers in Oncology	25	Q2	3.5	Radiother Oncol	564	Q1
Biomedical Signal Processing and Control	21	Q1	4.9	Proc CVPR IEEE	500	-
Computers in Biology and Medicine	20	Q1	6.7	Gynecol Oncol	447	Q1
Cancers	15	Q1	4.9	Lect Notes Comput Sci	408	-
Physics in Medicine And Biology	15	Q1	3.4	Sci Rep-UK	402	Q3
Journal of Applied Clinical Medical Physics	14	Q3	2.3	IEEE T Med Imaging	336	Q1
Medical Physics	14	Q1	3.9	CA-cancer J Clin	333	Q1
Gynecologic Oncology	10	Q1	4.5	IEEE Access	331	Q2

IF, impact factor; JCR, Journal Citation Reports.

Among the top 10 cited papers, four focus on AI screening and diagnosis, three on classification, two on radiotherapy, and one on detecting cancer cells. This diverse citation pattern indicates a comprehensive exploration of cervical cancer AI, covering diagnostic models, classification methods, and clinical applications.

### Journal analysis

A total of 334 journals have contributed to the literature, with 26 journals publishing five or more articles. Key journal indicators, including publication count, citation frequency, 5-year impact factor [IF(5)], and JCR category, are summarized in Table 3. Understanding these metrics aids researchers in identifying where to publish and understanding the field's dynamics.

The top three journals by publication quantity—“Diagnostics,” “Scientific Reports,” and “Frontiers in Oncology”—together published 25 papers, indicating their active engagement in cervical cancer-related AI. All top ten journals, except the “Journal of Applied Clinical Medical Physics,” are in the JCR Q1 and Q2 categories.

Despite “Medical Physics” not being the highest in publication count, it has the most citations (644), underlining its authoritative status. Both “Int J Radiat Oncol” and “Radiotherapy and Oncology” are recognized as leading journals with high impact factors and citation counts, reflecting their prominent roles in the fields of radiation oncology and medical physics.

This detailed analysis of journals highlights a lack of direct correlation between publication numbers and citation counts, suggesting that high-quality, impactful research is not solely defined by quantity.

### Keyword co-occurrence analysis

**Figure 4:**
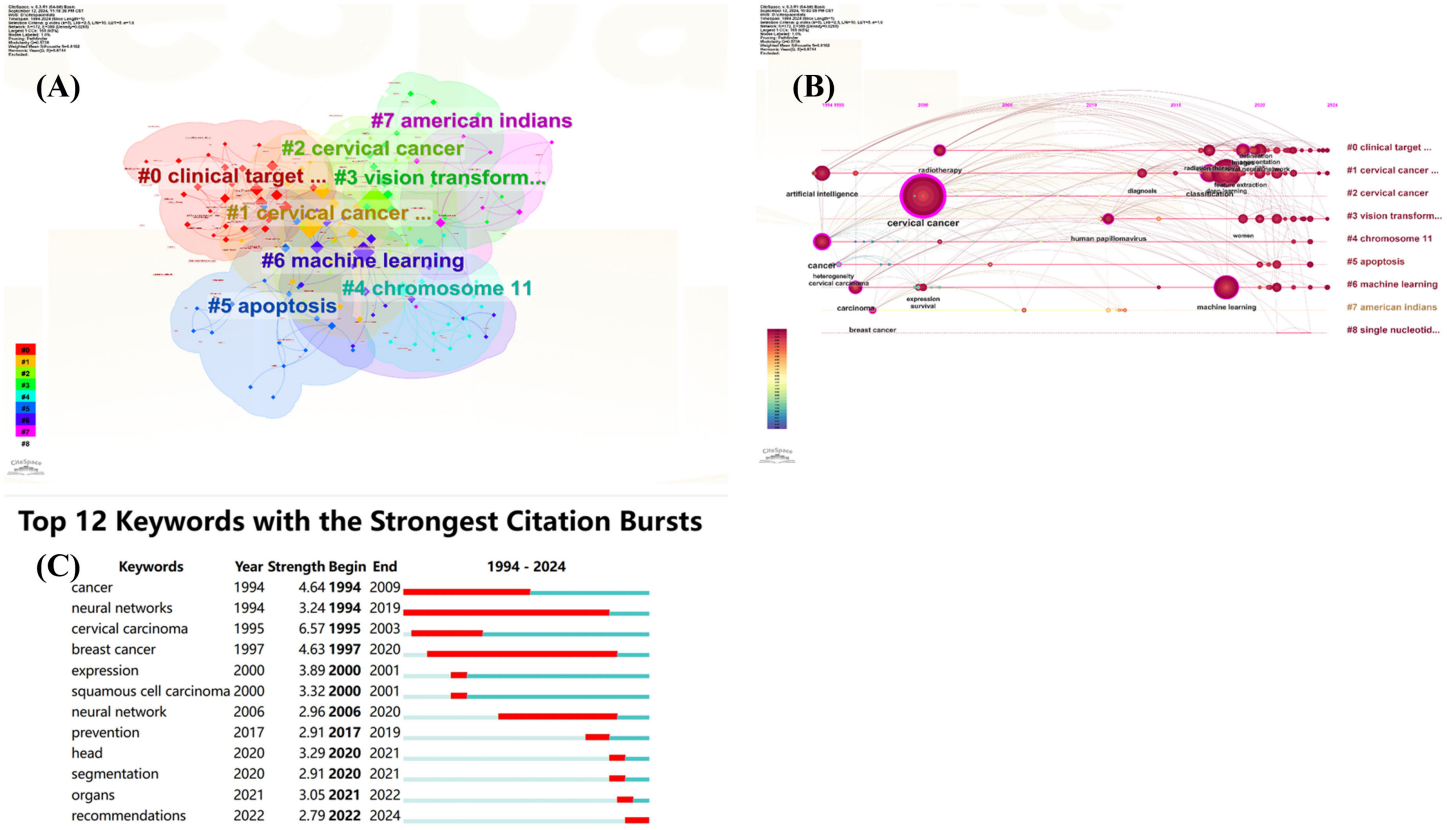
Visual map of the keyword network. **(A)** Keyword cluster analysis; **(B)** Timeline view of keywords clustering analysis on artificial intelligence for cervical cancer; **(C)** The top 12 keywords with the strongest citation bursts.

This study conducted a bibliometric analysis of cervical cancer-related AI literature, consisting of 2,787 keywords. Among these, 37 keywords appeared more than 20 times, forming eight distinct clusters (see Figure 4A), which illustrate the diverse research topics and interconnectedness within the field.

The cluster timeline view and co-occurrence analysis (shown in Figures 4B, C) reveal that “neural networks” emerged in 1994 and became prominent until 2019. This trend indicates the early onset and sustained popularity of AI in this domain. Recently, keywords such as “machine learning,” “segmentation,” “classification,” and “radiotherapy” have surged in frequency, suggesting they represent current hotspots in cervical cancer research, encompassing aspects like screening, diagnosis, treatment, and prognostic prediction.

“Cervical cancer” stands as the central focus of all research efforts, underscored by related terms such as “carcinoma,” which highlights its pathological characteristics. The frequent occurrence of “classification” indicates that accurate classification remains a crucial research direction, vital for precise diagnosis, personalized treatment planning, and prognosis evaluation.

The overarching term “artificial intelligence” symbolizes the core technological foundation supporting the research, introducing novel methodologies for diagnosing, treating, and investigating cervical cancer. Key branches, including “machine learning” and “deep learning,” enhance various applications such as image analysis, pathological diagnosis, and risk prediction, serving as powerful tools for early detection and effective treatment of cervical cancer.

Combining “radiotherapy” with segmentation techniques further optimizes treatment planning and accurately delineates lesions, enhancing therapeutic outcomes while minimizing side effects.

## Discussion

Cervical cancer is a malignant tumor that poses a significant threat to women's health and has gained increasing attention in recent years. The rapid advancement of AI technology has positioned its application in cervical cancer research as a notable area of interest. The distribution of publication years indicates a steep increase in research papers on cervical cancer and AI, with 86% published in the last 5 years. This trend underscores a growing interest in the field, heightened research investment, and an influx of researchers.

The correlation between the number of publications and citations aligns with trends in AI development, highlighting a shift in research focus from early screening and precise diagnosis to optimizing treatment strategies. Various studies leverage flexible AI algorithms, particularly deep learning and machine learning. Furthermore, there is a noticeable increase in studies utilizing multiple samples and centers, enhancing the reliability and generalizability of results. AI's role in auxiliary diagnosis and prognostic evaluation is becoming increasingly prominent in clinical applications.

Tracing the roots of AI in cervical cancer research reveals its inception in 1994, when Kok, MR, first applied neural networks for computer-assisted cervical cancer screening (16) and implemented this technology in 1996 (17). Progress was relatively slow for a time, but recent advancements in AI, particularly deep learning, have catalyzed significant growth in this research area. Since 2020, the number of researchers has surged, with prominent figures like Schiffman leading the field through a high volume of publications and citations. Similarly, Antani Sameer has made remarkable contributions, boasting an H-index of 36, which underscores his influence. Additionally, BC value analysis highlights key scholars such as Unknown, Jamal A., Ronneberger O., and He Km, whose contributions facilitate academic communication and collaboration, effectively promoting knowledge dissemination among research teams.

In summary, as AI continues to evolve, its integration into cervical cancer research offers promising potential for enhancing diagnostic accuracy and treatment optimization. Future research should focus on leveraging these advancements to translate them into practical clinical applications, ultimately improving patient outcomes.

Building on the previous analysis, we now explore multiple research hotspots in the field of AI related to cervical cancer, reflecting the diversity and depth of this area.

Firstly, the prominence of “neural networks” since 1994 indicates a long-standing and persistent interest in the application of AI in this field. Notably, “classification” has emerged as a key focus, crucial for achieving accurate diagnosis, tailoring personalized treatment plans, and evaluating prognosis. Machine learning and deep learning methods are receiving significant attention, as they employ various algorithms for applications such as image analysis, pathological diagnosis, and risk prediction. Recent studies have demonstrated the successful application of the random forest algorithm in matched case-control studies, highlighting its advantages in handling complex data and feature selection (18).

Furthermore, some researchers have developed multi-task learning models that can predict essential parameters pre-surgery, including staging, histology, grading, and lymph node metastasis (12, 19, 20). This model enhances efficiency and reduces the complexity of data reuse and model construction, illustrating the potential of integrating multiple algorithms in this field for more accurate classification of cervical cancer and the creation of comprehensive diagnostic and predictive models.

In addition, AI integrates with radiotherapy and segmentation technology. For instance, Bordigoni et al. (21) conducted a comprehensive evaluation of automatic segmentation models in pelvic radiotherapy, providing a valuable reference for clinical method selection. Accurate dose prediction and optimization are beneficial for improving the safety and effectiveness of radiotherapy while minimizing harm to normal tissues. Researchers have employed deep learning to investigate the appropriate radiation dose for cervical cancer (22, 23) and used deep convolutional neural networks and transfer learning to predict the potential harm of radiotherapy to the rectal area (24). The prominence of the keywords “radiotherapy” and “segmentation” reflects ongoing efforts to enhance treatment outcomes for cervical cancer while reducing side effects.

From the perspective of research focus, “cervical cancer” serves as the core topic, clearly defining the disease category, while “carcinoma” further delineates the pathological nature of cervical cancer. Over the past 5 years, keywords such as “machine learning,” “segmentation,” “classification,” “radiotherapy,” and “prevention” have frequently emerged, indicating that screening, diagnosis, treatment, and prognosis prediction are currently the most popular research areas within cervical cancer-related AI. Personalized therapeutic decision support exerts profound impacts on cervical cancer care practices. Machine learning models significantly enhance treatment individualization by integrating multimodal data (radiomics, genomics, and clinical parameters). Large language models (LLMs) like ChatGPT demonstrate the capacity to generate guideline-compliant, patient-tailored therapeutic recommendations (25). While current AI systems cannot substitute multidisciplinary expert teams, they demonstrate potential as decision-support adjuncts by not only rapidly aligning with clinical guidelines but also generating actionable recommendations for subsequent management and follow-up, ultimately enhancing clinical outcomes (25, 26). These research hotspots underscore that scholars in this domain are conducting comprehensive and in-depth investigations into various aspects of AI in cervical cancer, covering everything from diagnostic models and classification methods to prognosis evaluation and clinical applications.

Future research needs to strengthen interdisciplinary cooperation further, integrating knowledge and technology from various fields to tackle challenges in cervical cancer research. For example, medical experts can provide clinical data and professional insights, computer scientists can develop advanced AI algorithms and software, and biologists can delve deeper into the pathogenesis and biomarkers of cervical cancer.

At the national level, China and the United States lead the contributions to literature on cervical cancer-related AI. China accounts for 32% of the total publications in this domain, significantly ahead of the United States at 16% and India at 10%. Collectively, these three countries contribute 58% of publications, underscoring their dominant role in shaping the field. Notably, China not only has the highest number of citations but also ranks first overall in citation volume, followed by the United States and India.

The bibliometric (BC) value analysis indicates that the United States holds the highest BC value, pointing to its significant advantages in research investment, talent reserves, and infrastructure, which facilitate effective integration of both domestic and international research resources. China's relatively high BC value underscores its growing importance and influence in this field, supported by a robust research team and ample clinical resources. Meanwhile, the United States enhances this field's research landscape with its advanced technologies and strong research capabilities. Although the global development trend in cervical cancer AI appears unbalanced, international cooperation remains crucial. Strengthening collaboration and exchanges among countries will help integrate resources and share research outcomes.

At the institutional level, several prominent Chinese universities and research organizations are leading the charge in cervical cancer-related AI research. Notably, eight of the top 10 institutions are based in China, with the Chinese Academy of Sciences leading in publications. This reflects China's strong focus on research in this field, facilitated by its top experts, comprehensive research facilities, and robust scientific capabilities. In addition to the Chinese Academy of Sciences, Southern Medical University and Huazhong University of Science and Technology have also made noteworthy contributions. However, an analysis of the institutional cooperation network reveals that international collaboration is limited and primarily occurs within the same country. To address key challenges in cervical cancer-related AI research, future efforts must emphasize inter-institutional collaboration through resource sharing and joint research initiatives.

In the realm of journals publishing research on AI for cervical cancer, “Diagnostics,” “Scientific Reports,” and “Frontiers in Oncology” have prominent publication numbers, suggesting they may serve as core journals in this field. Additionally, journals such as “Medical Physics,” “International Journal of Radiation Oncology,” and “Radiotherapy and Oncology” are influential within radiation oncology, evidenced by their high citation totals. Given the multidisciplinary nature of AI, relevant research may also appear in journals outside traditional medical fields.

Citation analysis illustrates the relationships among scholarly works, reflecting knowledge flow and academic influence in cervical cancer-related AI. Current research priorities in cervical cancer management focus on four key fields: (i) deep learning-based cell classification and detection (15, 16), (ii) hybrid feature fusion techniques (6, 27), (iii) applications of transfer learning in predicting radiotherapy toxicity (24) and survival outcomes (28), and (iv) precision optimization of radiation therapy protocols (29). These approaches have demonstrated significant advancements in improving the accuracy of cervical cancer screening, diagnostic efficiency, and therapeutic efficacy. Notably, innovations such as Convolutional Neural Network (CNN) frameworks have achieved classification accuracy exceeding 95% (15). Treatment planning driven by deep reinforcement learning has demonstrated considerable improvements. Hybrid methodologies that integrate artificial feature engineering with deep architectures, exemplified by DeepCervix, have further optimized interpretability while sustaining high performance, with classification accuracy ranging from 90.32 to 99.85% (6). These strategies provide robust technical support for clinical decision-making, serving as critical foundations for personalized oncology management.

Ultimately, the goal of AI research in cervical cancer is to improve clinical diagnosis and treatment. Future efforts should focus on translating AI technologies into practical clinical applications, such as user-friendly diagnostic tools and treatment decision support systems, to enhance medical professionals' efficiency and accuracy. Furthermore, rigorous evaluation of AI safety and reliability is essential to ensure its effectiveness in clinical practice.

## Limitations

This bibliometric analysis on AI applications in cervical cancer reveals certain limitations. Firstly, our focus on English-language literature from the SCIE and ESCI indexes of the WOSCC database may omit valuable insights from other languages and sources. Additionally, the study primarily examined evolving patterns based on keywords and references while neglecting factors like funding sources that could significantly impact research progress.

Moreover, the analysis may overlook emerging topics, such as the relationship between cervical cancer AI research and patient mental health. Finally, issues like data loss and author name errors during bibliometric processes can compromise accuracy.

To enhance this research, we recommend broadening the database to include more diverse literature, analyzing non-English sources, manually curating keywords for accuracy, and improving the reliability of bibliometric tools, ultimately leading to better clinical guidance.

## Conclusion

This bibliometric analysis offers an overview of the research status and trends in cervical cancer AI. The potential applications of AI are broad—spanning auxiliary screening, diagnostic support, treatment optimization, and prognostic prediction—yet significant challenges remain. While China and the United States lead in research, other nations are intensifying their efforts to catch up.

To further develop AI technology in cervical cancer, fostering interdisciplinary and international collaboration is crucial. We envision that as technology and research advance, AI will deliver increasingly effective solutions for cervical cancer prevention and treatment.

## Data Availability

The raw data supporting the conclusions of this article will be made available by the authors, without undue reservation.
